# Smart Thermosensitive Mesoporous Silica Composites for Precisely Triggered Linalool Release

**DOI:** 10.1021/acsomega.6c04870

**Published:** 2026-10-28

**Authors:** Zhenhui Gao, Yanhua Lu, Fengfeng Zhang, Wenlong Bao, Xinhua Song, Fanyu Kong, Rongning Liang, Dean Song

**Affiliations:** † State Agriculture Ministry Laboratory of Quality & Safety Risk Assessment for Tobacco, Tobacco Research Institute, Chinese Academy of Agricultural Sciences, Qingdao 266101, China; ‡ China Tobacco Shandong Industrial Co., Ltd., Jinan 250014, China; § Shandong Key Laboratory of Coastal Zone Environmental Processes and Ecological Security, Yantai Institute of Coastal Zone Research, 74620 Chinese Academy of Sciences, Yantai, Shandong 264003, P. R. China

## Abstract

To overcome the application limitations of linalool, including its high volatility and poor stability, mesoporous silica MCM-41 was used as the carrier, and *N*-isopropylacrylamide (NIPAM) was employed as the thermosensitive monomer in this study. Acrylamide (AM) was introduced to modulate the lower critical solution temperature (LCST) of poly­(*N*-isopropylacrylamide) (PNIPAM), while *N*,*N*′-methylenebis­(acrylamide) (BIS), used as a cross-linking agent, was added to enhance structural stability, thus constructing the MCM-41-PNIPAM thermosensitive composite sustained-release system. Characterization results confirmed the successful synthesis of the target material, whose LCST increased to 82–85 °C with the increasing AM doping ratio. Sustained-release experiments indicated that the release rate of linalool significantly increased when the environmental temperature exceeded the LCST. Antibacterial tests revealed that the composite exhibited a remarkable inhibitory effect on *Bacillus subtilis*, *Staphylococcus aureus*, and *Escherichia coli* following the temperature-triggered release of linalool. Moreover, the antibacterial effect could be precisely controlled by regulating the ambient temperature, thereby modulating the release. This study presents a new strategy for the stabilized application of linalool, achieves an intelligent response effect of “temperature-triggered and on-demand release,” and offered a universal method for constructing intelligent preservation systems.

## Graphical abstract

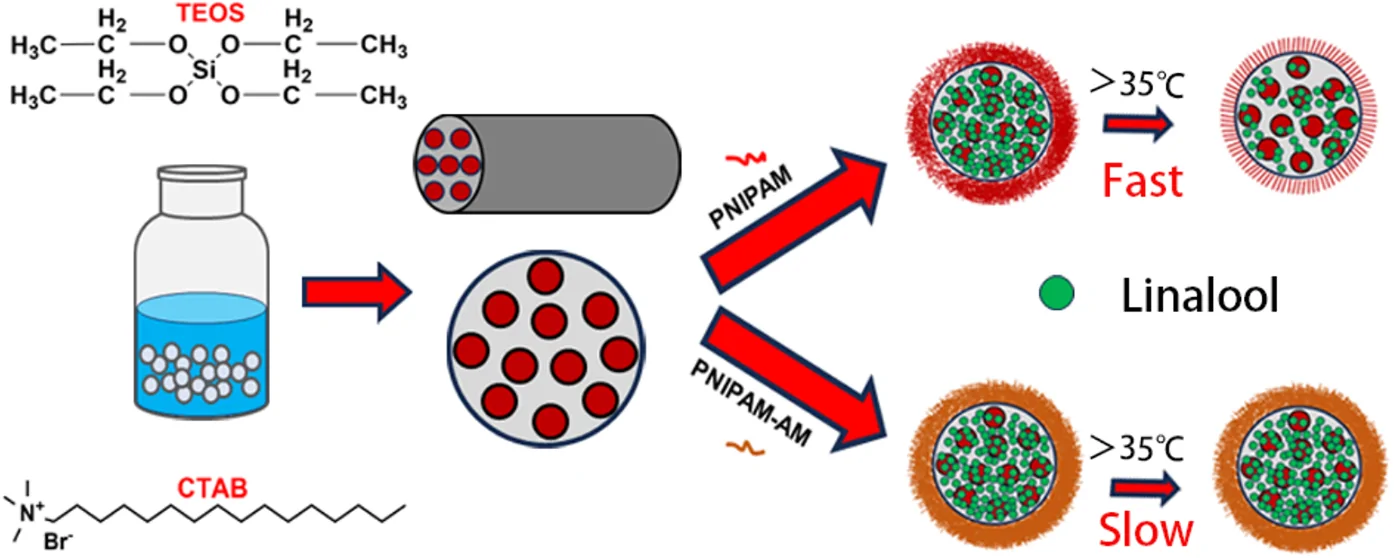

## 1 Introduction

Linalool (2,6-dimethyl-2,7-octadien-6-ol) is an acyclic monoterpene alcohol that naturally occurs in a variety of aromatic plants. Because of its unique aroma, linalool is widely used in perfumes, household cleaners, and cosmetic products (shampoo, bath products, soap, lotion, face cream, antiperspirant, and hair spray). Furthermore, linalool has been designated as Generally Recognized as Safe (GRAS) by the U.S. Food and Drug Administration (FDA), supporting its potential application as a food-compatible antimicrobial agent in food preservation systems.^1–4^ Linalool has been reported to exhibit broad-spectrum antimicrobial activity against bacteria and fungi, including *Escherichia coli*, *Salmonella enterica*, *Staphylococcus aureus*, *Listeria monocytogenes*,^5^ *Acinetobacter baumannii*,^6^ *Pseudomonas aeruginosa*,^7^ *Proteus mirabilis*,^8^ *Candida albicans*,^9^ *Microsporum canis*, and *Microsporum gypseum*.^10^ The mechanism of linalool primarily involves disruption of microbial cell membrane integrity and inhibition of bacterial and fungal biofilm formation, thereby suppressing microorganism growth.^11–13^ Nevertheless, its high volatility, poor stability, short action duration, and strong pungent odor limit its direct application in food preservation.^14^

Current studies have adopted encapsulation strategies (e.g., microcapsules and liposomes) to improve the stability and bioavailability of linalool. Nevertheless, the lack of intrinsic stimulus responsiveness (e.g., temperature, pH, redox-triggered mechanisms) in these carriers hinders the realization of on-demand release kinetics.^15,16^ This limitation not only facilitates premature linalool leakage during storage but also compromises its bioactivity in practical applications (such as antimicrobial food coatings) because of insufficient payload delivery. Consequently, it severely restricts the implementation potential in scenarios requiring spatiotemporal control.

To address the challenges of controlled release and stability of active substances, stimulus-responsive polymers have emerged as a major research focus, owing to their unique environmental stimulus responsiveness. These polymers undergo dramatic variations in the swelling ratio when exposed to external triggers including temperature, pH, solvent composition, and electric field strength, with promising applications in biomedical engineering, stimulus-responsive separation, and soft electronics.^16–18^ Among various triggers, temperature is the most widely adopted stimulus in stimulus-responsive polymer systems due to its ease of control.^19–21^ Thermosensitive polymers represented by poly­(*N*-isopropylacrylamide) (PNIPAM) undergoes a characteristic phase transition in the aqueous solution when heated above the lower critical solution temperature (LCST). As the temperature rises, hydrogen bonds between polymer chains and water molecules break, causing hydrated polymer segments to collapse into hydrophobic domains. This structural shift forms a diffusion barrier that facilitates controlled release of encapsulated payloads.^22,23^

However, organic compounds generally suffer from inherent drawbacks in mechanical properties, whereas inorganic materials feature outstanding chemical stability and mechanical strength. By fabricating organic–inorganic composite systems, researchers can greatly enhance the chemical stability and mechanical performance of organic matrices via the reinforcement provided by the inorganic phase.^24^ In recent years, mesoporous silica nanoparticles, which possess tailorable pore structures, high specific surface areas, and excellent biocompatibility, have been widely used as an inorganic matrix in organic–inorganic composites and are recognized as promising carrier for sustained drug delivery.^25–27^ On this basis, the poly­(*N*-isopropylacrylamide)-mesoporous silica (PNIPAM–SiO_2_) composite system has been regarded as an effective carrier platform to overcome the drawbacks of single-component thermosensitive carriers and improve controlled release behavior, making it an active research area in thermosensitive sustained delivery systems.

Despite the advantages of the PNIPAM–SiO_2_ composite, its application in physiological environments is limited by the LCST of approximately 32 °C.^17^ This temperature range cannot satisfy the demands of food preservation scenarios requiring high-temperature-triggered release mechanisms, such as hot-fill sterilization of pasteurized foods,^28^ microwave-induced activation of antimicrobial properties in ready-to-eat foods,^29^ and emergency protection against extremely high temperatures during cold-chain transportation.^30^ Although existing studies^31–33^ have regulated the LCST of PNIPAM by introducing polar groups (e.g., carboxyl, amide, hydroxyl), research on modifying PNIPAM–SiO_2_ composites for linalool encapsulation remains scarce. Moreover, no universal strategy to balance stability, stimuli responsiveness, antibacterial activity, and precise temperature-triggered release has yet to be developed.

Therefore, this study aims to develop a modification strategy by incorporating specific hydrophilic functional groups into PNIPAM–SiO_2_ composites to regulate their thermosensitive behavior in an intelligent and demand-oriented manner and increase the LCST to a temperature range suitable for high-temperature food preservation applications. Meanwhile, a linalool-loaded PNIPAM–SiO_2_ composite system was fabricated and systematically evaluated in terms of storage stability, thermoresponsiveness, antibacterial activity, and temperature-triggered release kinetics. The developed composite is designed as a functional antimicrobial component for active food packaging systems, including antimicrobial coatings or packaging films, rather than as a direct food additive.^34,35^ Through temperature-responsive regulation of linalool release, this system aims to enhance the utilization efficiency and preservation performance of linalool under temperature-variable food-storage and -processing conditions. This work provides a novel strategy for developing smart controlled release materials toward advanced active food packaging applications.

## 2 Experimental Section

### 2.1 Chemicals and Materials

Fluorescent stains SYTO 9 and propidium iodide (PI) were purchased from Sigma-Aldrich. Tetraethyl orthosilicate (TEOS), poly­(ethylene oxide)-poly­(propylene oxide)-poly­(ethylene oxide) triblock copolymer (P123), cetyltrimethylammonium bromide (CTAB), *N*-isopropylacrylamide (NIPAM), acrylamide (AM), *N,N′*-methylenebis­(acrylamide) (BIS), 3-(trimethoxysilyl)­propyl methacrylate (MPS, KH-570), concentrated hydrochloric acid (HCl), aqueous ammonia (NH_3_·H_2_O), potassium persulfate (KPS), absolute ethanol (C_2_H_5_OH), glacial acetic acid (CH_3_COOH), and linalool were purchased from Shanghai Aladdin Biochemical Technology Co., Ltd. All reagents were of analytical grade and used as received without further treatment. *Bacillus subtilis* (BS), *S. aureus* (SA), and *E. coli* (EC) were purchased from Shanghai Jizhi Biochemical Technology Co., Ltd. Deionized water was prepared using a Pall Cascada laboratory ultrapure water system, with a resistivity of 18.2 MΩ·cm.

### 2.2 Synthesis of Mesoporous Silica

The synthesis of SBA-15 and MCM-41 was performed according to previously reported methods with minor modifications.^36,37^ For SBA-15 synthesis, 4.2 g of P123 was dissolved in 150 mL of 2.0 M HCl solution under stirring at 45 °C for 24 h, followed by the dropwise addition of 9.8 mL TEOS. The mixture was further stirred at 45 °C for 15 h and statically aged at 80 °C for 48 h. The obtained solid was collected, washed with deionized water, dried at 80 °C for 12 h, and calcined at 550 °C for 4 h (5 °C/min) to remove the template.

For MCM-41 synthesis, 12 mL ammonia solution, 50 mL deionized water, and 50 mL ethanol were mixed, followed by addition of 2.4 g CTAB. After stirring for 15 min, 3.4 g TEOS was added dropwise, and the mixture was reacted at room temperature for 2 h. The precipitate was collected, washed, dried at 90 °C for 12 h, and calcined at 550 °C for 4 h (5 °C/min) to obtain template-free MCM-41.

### 2.3 Synthesis of Thermosensitive Polymers MCM-41-PNIPAM

The thermosensitive composite carrier MCM-41-PNIPAM was synthesized through three sequential steps: acidification of MCM-41, KH-570 silane modification, and in situ copolymerization of thermosensitive monomers according to a previously reported method with modifications.^38^

First, MCM-41 (500 mg) was dispersed in 20 mL of 1 M HCl solution and refluxed at 80 °C for 2 h. The acid-treated silica was collected, washed with deionized water until neutral, and dried at 80 °C for 12 h. Subsequently, 1 g of acidified MCM-41 was dispersed in 60 mL of absolute ethanol and ultrasonicated for 40 min. The pH was adjusted to 4 using acetic acid, followed by nitrogen purging for 30 min. KH-570 (1 mL), diluted with 10 mL ethanol, was added dropwise, and the mixture was reacted at 80 °C for 4 h. The obtained KH-570-modified MCM-41 was collected, washed with ethanol, and vacuum-dried at 60 °C for 12 h.

For the preparation of MCM-41-PNIPAM, KH-570-modified MCM-41 was dispersed in deionized water and ultrasonicated for 1 h. NIPAM, AM, and BIS were subsequently added according to the compositions listed in Table 3. After further dispersion, the mixture was stirred at 300 rpm and 60 °C for 2 h. The polymerization was initiated by adding the initiator solution under N_2_ protection, followed by reaction at 80 °C for 5 h. The final product was collected by centrifugation, washed alternately with deionized water and ethanol, and vacuum-dried at 80 °C for 12 h to obtain thermosensitive MCM-41-PNIPAM composite nanoparticles with different monomer ratios.

### 2.4 Linalool Loading

0.3 g of MCM-41-PNIPAM nanoparticles were dispersed in 20 mL absolute ethanol and ultrasonicated for 30 min. One milliliter of linalool ethanol solution was added, followed by 30 min of ultrasonication. The mixture was transferred to a 50 mL three-neck flask and stirred at 25 °C for 1 h. After heating to 80 °C under reflux for 24 h, the product was cooled, centrifuged, and washed with ethanol to remove unbound linalool. Vacuum drying at 30 °C for 24 h yielded MCM-41-PNIPAM–linalool composite powder.

### 2.5 Characterization

Surface morphology was analyzed using scanning electron microscopy (SEM, Hitachi S-4800) at an accelerating voltage of 5 kV. Prior to imaging, the samples were sputter-coated with a thin layer of gold to prevent charging effects. Fourier transform infrared (FTIR) spectra were recorded on a Thermo Nicolet iS10 spectrometer equipped with an attenuated total reflectance (ATR) accessory over the wavenumber range of 400–4000 cm^–1^ with 32 scans at a resolution of 4 cm^–1^. Elemental analysis of carbon and nitrogen contents in the samples was performed using a Vario Micro elemental analyzer. Approximately 15 mg of accurately weighed, dried sample was wrapped in a tin boat and loaded into the combustion furnace at 1150 °C under a helium carrier gas flow. Loading efficiency and LCST were determined via thermogravimetric analysis (TGA) and differential scanning calorimetry (DSC) using an STA449F3 instrument: TGA was conducted at 5 °C/min from 20 to 700 °C and DSC at 1 °C/min from 20 to 120 °C. Nitrogen adsorption–desorption isotherms were measured at −196 °C using a Micromeritics ASAP 2460 analyzer. Prior to measurement, all samples were degassed at 110 °C for 4 h under vacuum. The specific surface area was calculated using the Brunauer–Emmett–Teller (BET) method in the relative pressure (*P*/*P*_0_) range of 0.05–0.25, and the pore size distribution was derived from the Barrett–Joyner–Halenda (BJH) method applied to the desorption branch.

### 2.6 Investigation and Evaluation of Linalool Sustained-Release Performance

Different mass ratios of MCM-41-PNIPAM–linalool composite carrier powder and pure linalool were weighed and placed in sample vials, and their initial masses were accurately recorded. Afterward, the vials were stored at different temperatures (30, 50, and 80 °C) to carry out controlled release tests. At predetermined time points, each group of samples was weighed to record the mass changes. The sustained-release efficiency of linalool was calculated based on the mass loss rate.

The release kinetics of linalool could be described by the following mathematical eqs 1 (zero-order), 2 (Higuchi), 3 (Ritger–Peppas), and 4 (first-order).^38,39^

1 $$\frac{M_{t}}{M_{0}}=\mathrm{Kt}$$

2 $$\frac{M_{t}}{M_{0}}=Kt^{0.5}$$

3 $$\frac{M_{t}}{M_{0}}=Kt^{n}$$

4 $$\frac{M_{t}}{M_{0}}=1-e^{-\mathrm{Kt}}$$

where *n* is the release index, and *K* represents the zero-order, Higuchi, Ritger–Peppas, first-order rate constants of the corresponding kinetic models, respectively.

### 2.7 Determination of Encapsulation Efficiency and Loading Efficiency

A linalool standard solution was prepared by dissolving an accurately weighed amount of linalool reference standard in absolute ethanol to obtain a stock solution (1.0 mg/mL). The stock solution was diluted to prepare working solutions of 4, 8, 12, 16, and 20 μg/mL. Ethanol was used as the blank, and a Beckman Coulter DU-800 spectrophotometer was employed to scan spectra in the 200–300 nm range for determination of the maximum absorption wavelength of linalool. The calibration curve was established by plotting absorbance against linalool concentration, and the corresponding regression equation and correlation coefficient were obtained.

For linalool quantification in the composite, a known amount of MCM-41-PNIPAM–linalool was dispersed in absolute ethanol and ultrasonicated to extract the loaded linalool from the mesoporous channels and polymer network. After centrifugation, the supernatant was collected, and the residue was repeatedly washed with ethanol. The combined extracts were diluted to a fixed volume, and the linalool concentration was calculated according to a calibration curve.

The encapsulation efficiency (EE) and loading efficiency (LE) of the linalool-loaded composite were calculated using eqs 5 and 6.

5 $$EE=\frac{m}{m_{0}}\times 100\%$$

6 $$\mathrm{LE}=\frac{m}{m_{1}}\times 100\%$$

where EE represents the encapsulation efficiency of the composite, m stands for the mass of entrapped linalool in the composite, and *m*_0_ refers to the initial mass of linalool added during synthesis. LE denotes the loading efficiency of the composite, and *m*_1_ is the total mass of the linalool-loaded composite.

All samples were measured in triplicate, and the average value and standard deviation were calculated to ensure accuracy and repeatability of the experimental data.

### 2.8 UV Resistance and Storage Stability of MCM-41-PNIPAM–Linalool

The samples underwent two aging treatments: continuous UV irradiation for 24 h and dark storage at ambient temperature for 15 days. For UV irradiation, the samples were placed in a UV cross-linking chamber equipped with an 8 W low-pressure mercury lamp emitting at 254 nm, with an intensity of 1.2 mW/cm^2^. The distance between the light source and the sample surface was fixed at 15 cm, and the chamber dimensions were 60 × 60 × 60 cm. For the storage stability assay, the samples were stored in airtight glass vials at ambient temperature (25 ± 2 °C) in the dark for 15 days. Following the aging treatments, the retained linalool was extracted from the samples using anhydrous ethanol. The concentration of linalool in the extracts was quantified via UV–vis spectrophotometry against a pre-established calibration curve, and the retention rate was calculated accordingly. Meanwhile, the antibacterial activity of the composites after aging was assessed using the plate colony-counting method.

### 2.9 Antibacterial Performance Testing and Antimicrobial Efficacy Analysis

The antibacterial activity of the prepared composites was evaluated using the spread-plate counting method. LB medium was prepared and sterilized by autoclaving. *B. subtilis*, *S. aureus*, and *E. coli* were cultured in LB medium at 37 °C overnight (120 rpm). The bacterial suspensions were collected, washed three times with sterile 0.9% NaCl solution, and adjusted to approximately 10^8^ CFU mL^–1^ using UV–vis spectrophotometry (OD_600_ = 0.15). The suspensions were subsequently diluted for antibacterial experiments.

For antibacterial evaluation, bacterial suspensions were mixed with different samples, including MCM-41, MCM-41-PNIPAM, MCM-41-PNIPAM–linalool, and free linalool. *B. subtilis*, *S. aureus*, and *E. coli* without any treatment were used as the control. After incubation, the mixtures were spread onto LB agar plates and cultured at 37 °C for colony observation and antibacterial activity evaluation.

The inhibitory activity of MCM-41-PNIPAM–linalool was evaluated using the disk diffusion method. Sterile filter paper disks were soaked in the test sample and then placed onto LB agar plates precoated with an activated bacterial suspension (OD_600_ = 0.15). Blank disks without any sample served as the negative control. After incubation at 37 °C, the diameters of the inhibition zones were measured.

The minimum inhibitory concentration (MIC) and minimum bactericidal concentration (MBC) of free linalool were determined using the broth 2-fold serial method. A series of sample concentrations ranging from 256 to 0.0625 mg/mL were prepared in LB liquid medium by 2-fold gradient dilution, followed by inoculation with activated bacterial suspension. Controls included culture medium alone and medium with bacterial suspension without the sample. All cultures were incubated at 37 °C with shaking at 120 rpm. The MIC was defined as the lowest concentration of the sample that resulted in no visible bacterial turbidity, as determined by naked-eye observation. For MBC determination, aliquots from the MIC and higher concentration groups were spread onto LB agar plates and incubated at 37 °C. The MBC was recorded as the lowest concentration that yielded fewer than 20 colonies on the plates.

To investigate the temperature-responsive antibacterial behavior of the linalool-loaded thermosensitive composite, CLSM combined with live/dead fluorescence staining was performed. The MCM-41-PNIPAM–linalool composite was incubated with *B. subtilis* (10^9^CFU mL^–1^) under different temperature conditions. After treatment, SYTO 9 (3.34 μM) and propidium iodide (20 μM) were added and incubated in the dark for 30 min. The stained bacterial cells were subsequently observed using CLSM (FV1000, Olympus, Japan).

### 2.10 Cytotoxicity Evaluation

The cytotoxicity assay was performed by using L929 murine fibroblast cells as the model cells for evaluation, which were kindly provided by Shandong International Biotechnology Park Development Co., Ltd. The cells were cultured in RPMI 1640 medium supplemented with fetal bovine serum and a mixture of penicillin and streptomycin and incubated at 37 °C under a 5% CO_2_ atmosphere. All procedures were conducted under aseptic conditions to prevent microbial contamination. Cells were seeded in 96-well plates. Gradient concentrations of MCM-41, MCM-41-PNIPAM, and MCM-41-PNIPAM–linalool were added separately, and the plates were incubated for 48 h. After 48 h, the cells were observed by using an inverted fluorescence microscope (Revolve FL, Echo Laboratories, USA). Additionally, cell viability was assessed using the Cell Counting Kit-8 (CCK-8) assay, with absorbances measured at 450 nm on a microplate reader (Elx808L, Biotek, USA).

## 3 Results and Discussion

### 3.1 Structural Characterization of Mesoporous Silica Supports and Their Influence on Linalool Loading Performance

To investigate the influence of mesoporous architecture on linalool loading behavior, SBA-15 and MCM-41 with distinct pore structures were selected as comparative silica supports. Both materials were synthesized using a soft-templating method, and their ordered mesoporous structures were confirmed by SEM and nitrogen adsorption–desorption analysis (Figure 1). The obtained materials exhibited typical Type IV isotherms, indicating well-developed mesoporosity. The structural parameters were consistent with those reported for standard SBA-15 and MCM-41, demonstrating the successful preparation of mesoporous silica supports.

**Figure 1:**
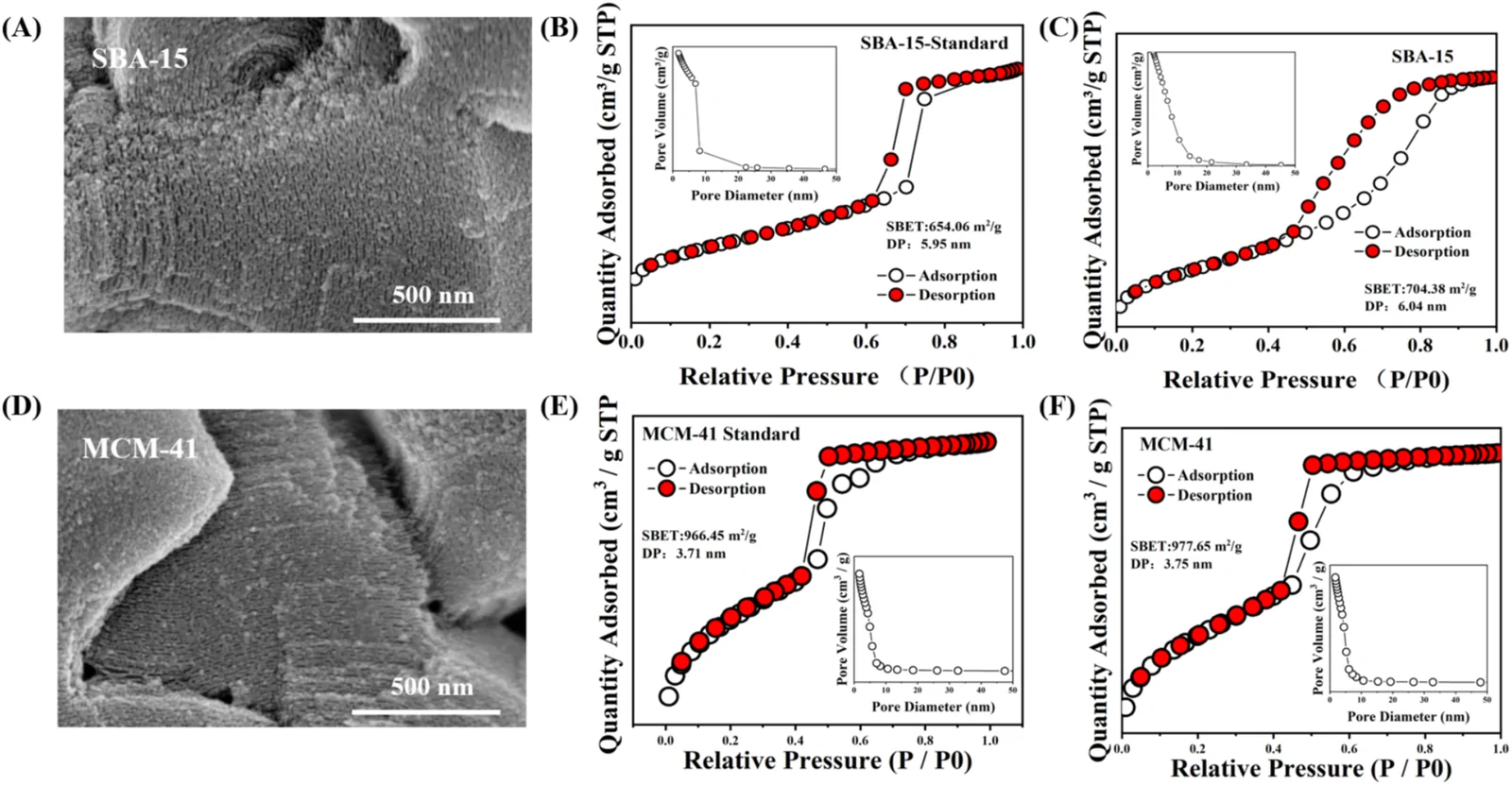
Morphology and pore structure characterization of mesoporous silica materials. (A, D) Scanning electron microscopy (SEM) images of SBA-15 standard and MCM-41 standard, respectively; (B, E) Nitrogen adsorption–desorption isotherms and pore size distributions of the standard SBA-15 and MCM-41 samples, respectively; (C, F) Nitrogen adsorption–desorption isotherms and pore size distributions of the as-synthesized SBA-15 and MCM-41 samples in this work, respectively.

The effect of the pore architecture on linalool loading was further evaluated through solvent-free physical grinding. As shown in Figure S1, increasing the linalool/support ratio induced different morphological changes in SBA-15 and MCM-41. SBA-15 exhibited gel-like aggregation at a lower linalool loading ratio (0.3 mL/0.1g), whereas MCM-41 retained a powder-like morphology and required a higher linalool amount (0.4 mL/0.1g) to form a gel state. This difference suggests that the smaller and more uniform pore channels of MCM-41 provide enhanced confinement for linalool molecules and reduce the level of excessive surface accumulation. Therefore, MCM-41 was selected as the optimal mesoporous support for subsequent thermoresponsive composite fabrication because of its superior loading compatibility and retention capability.

### 3.2 Fabrication and Performance Evaluation of the MCM-41-PNIPAM–Linalool Composite

To verify the successful fabrication of MCM-41-PNIPAM–linalool, the samples were comprehensively characterized by a combination of SEM, BET, CHNS, FTIR, and TGA.

As shown in Figure S2, bare MCM-41 exhibited aggregated nanoscale spherical particles with relatively uniform morphology and abundant interparticle pores. After copolymerization with NIPAM, AM, and BIS, followed by linalool loading, the surface morphology of MCM-41 was significantly altered. The originally smooth spherical particles became irregular, and the polymer modification resulted in a more compact aggregate structure, indicating successful surface functionalization.

Nitrogen adsorption–desorption measurements were performed to investigate the pore characteristics of the samples (Figure S3). All samples exhibited typical Type IV isotherms with H1 hysteresis loops, demonstrating that the ordered mesoporous structure of MCM-41 was retained after modification. Compared with bare MCM-41, polymer grafting caused decreases in specific surface area, pore size, and pore volume. After linalool loading, these parameters further decreased, indicating partial filling of the mesoporous channels by linalool. These results confirm the successful construction of the polymer-functionalized linalool-loaded MCM-41 composite.

Elemental analysis (Table 1) further confirmed the successful modification and linalool incorporation. Bare MCM-41 exhibited negligible carbon and nitrogen contents (0.152 and 0.020%, respectively). After grafting with PNIPAM, the carbon and nitrogen contents increased substantially to 20.420 and 6.422%, respectively. Following linalool loading, the carbon content further increased to 38.085%, whereas the nitrogen content decreased to 4.455% because of the nitrogen-free nature of linalool. These results verify the successful incorporation of both the polymer component and linalool into the MCM-41-based composites.

**Table 1:** Total Carbon and Nitrogen Content Ratios of MCM-41, MCM-41-PNIPAM, and MCM-41-PNIPAM–Linalool

**samples**	**carbon %**	**nitrogen %**
MCM-41	0.152	0.020
MCM-41-PNIPAM	20.420	6.422
MCM-41-PNIPAM-linalool	38.085	4.455

As shown in Figure 2, the FTIR spectrum of the MCM-41-PNIPAM–linalool composite exhibited several characteristic absorption bands. The characteristic amide I (C=O stretching) and amide II (N–H bending) bands of the PNIPAM copolymer appeared at 1643 and 1545 cm^–1^, respectively. In addition, absorption bands attributable to linalool were observed around 3400, 1640, and 1000–1100 cm^–1^, corresponding to O–H stretching, C=C stretching, and C–O stretching vibrations, respectively. The coexistence of these characteristic bands indicates the successful grafting of the thermoresponsive copolymer onto MCM-41 and the successful incorporation of linalool into the composite.

**Figure 2:**
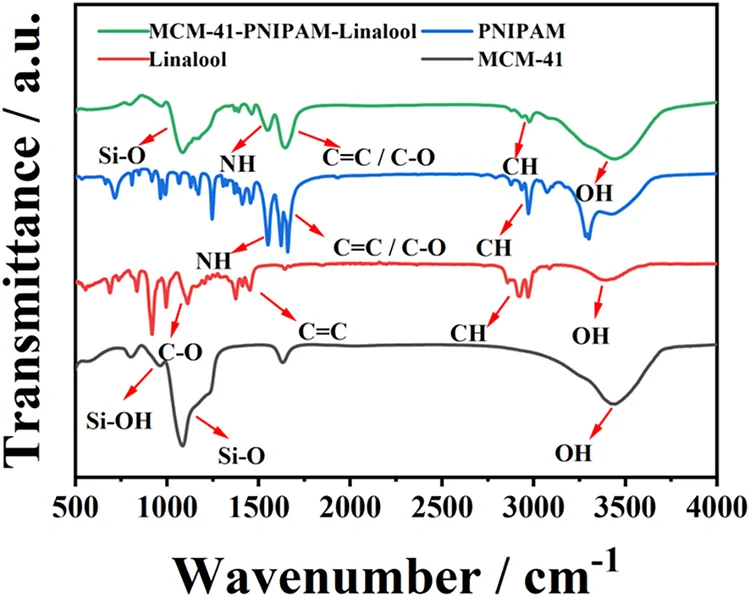
Comparison of FTIR spectra of MCM-41, PNIPAM, linalool and the target composite MCM-41-PNIPAM–linalool.

Thermogravimetric analysis was conducted to evaluate the thermal stability of all samples (Figure 3). Bare MCM-41 exhibited a negligible weight loss, mainly due to the removal of adsorbed water and surface silanol condensation. Pure linalool volatilized rapidly upon being heated. In contrast, MCM-41-PNIPAM displayed two weight loss stages associated with water evaporation and copolymer degradation. The linalool-loaded composite exhibited greater weight loss, further confirming successful linalool loading and its improved thermal retention within the polymer-functionalized mesoporous structure.

**Figure 3:**
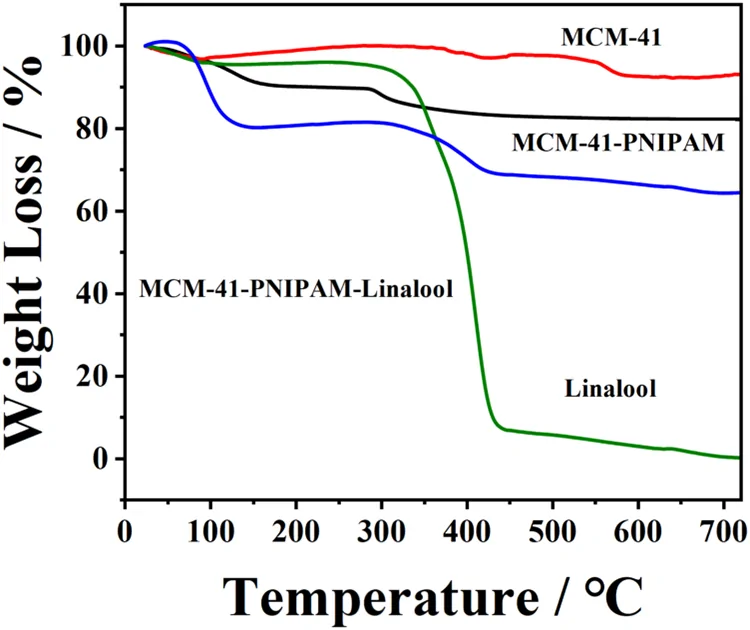
Comparison of TGA spectra of MCM-41, MCM-41-PNIPAM, linalool and the target material MCM-41-PNIPAM–linalool.

UV–vis spectrophotometry was used to determine the encapsulation efficiency and loading efficiency of linalool in MCM-41-PNIPAM. The calibration curve established from the UV–vis measurements is shown in Figure S4. The corresponding EE and LE values are summarized in Table 2. The composite exhibited an EE of 11.58 ± 0.49% and an LE of 35.23 ± 0.53%, confirming the successful loading of linalool into the polymer-functionalized mesoporous carrier.

**Table 2:** Encapsulation Efficiency and Loading Efficiency of MCM-41-PNIPAM–Linalool

**sample**	***m***_**0**_	***m***_**1**_	**EE**	**LE**
MCM-41-PNIPAM–linalool	0.3 g	0.1 g	11.58 ± 0.49%	35.23 ± 0.53%

### 3.3 AM Copolymerization-Regulated LCST of Thermoresponsive Copolymers and Temperature-Responsive Linalool Release Behavior of the Composite Carrier

PNIPAM is one of the most extensively studied thermosensitive polymers with an LCST of approximately 32 °C. It has been reported that copolymerization with hydrophilic monomers can effectively regulate its volume phase transition temperature (VPTT).^16^ In this study, AM was selected as the comonomer to modulate the VPTT of the copolymer shell. DSC was employed to investigate the thermosensitive responsiveness of MCM-41-PNIPAM–linalool nanoparticles with different AM/NIPAM doping ratios (Figure S5). As shown in Table 3, precise modulation of the thermosensitivity of P­(NIPAM-SiO_2_-AM-BIS) was successfully realized by introducing the hydrophilic monomer AM. With fixed dosages of NIPAM and SiO_2_, the LCST of the polymer increased significantly from 31 to 33 °C to approximately 82–85 °C as the doping amount of AM was gradually raised. This phenomenon is primarily attributed to the strong hydrophilicity of the amide groups in AM molecules. Their incorporation enhances hydrogen bonding between the polymer chains and water molecules, thus improving improves the hydration capacity of the polymer. Consequently, hydrophobic collapse during heating is delayed, effectively expanding the thermosensitive range.^40^ It should be noted that the LCST of 82–85 °C presented here serves as a representative demonstration of the tunable range of our system, rather than a fixed target for all applications. By adjusting the AM content, the LCST can be precisely tailored to meet various temperature requirements, such as hot-fill pasteurization, microwave-assisted thermal processing, or even lower-temperature scenarios for different food preservation needs. This flexibility underscores the universal adaptability of the proposed platform.

**Table 3:** Formulations and Corresponding LCST of MCM-41-PNIPAM Nanoparticles with Different AM Doping Amount

**sample**	**SiO**_**2**_ **/mg**	**NIPAM/mg**	**AM/mg**	**BIS/mg**	**KPS/mg**	**LCST/°C**
A	1000.00	600.00	0.00	40.52	20.00	31.00–33.00
B	1000.00	600.00	41.50	45.02	21.39	39.00–46.00
C	1000.00	600.00	93.38	50.65	23.11	48.00–61.00
D	1000.00	600.00	160.08	57.88	25.34	73.00–74.00
E	1000.00	600.00	251.25	68.13	28.38	82.00–85.00

To systematically elucidate the temperature-responsive sustained-release mechanism of linalool from thermosensitive composite carriers, the in vitro release behaviors of free linalool and the composite carriers (A and D) were comparatively evaluated at 30, 50, and 80 °C. The corresponding release profiles are presented in Figure 4. Overall, free linalool exhibited a typical burst-release behavior regardless of temperature, whereas both composite carriers effectively prolonged linalool release through the combined effects of PNIPAM thermoresponsiveness and mesoporous confinement. Owing to their different LCST, Groups A and D displayed distinct temperature-dependent release characteristics.

**Figure 4:**
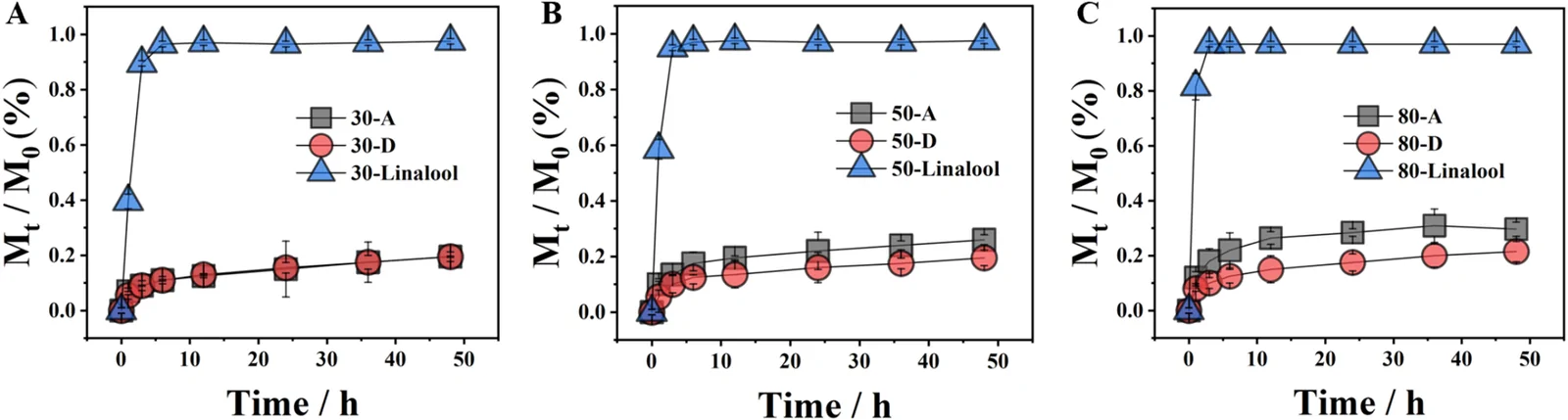
Weight change curves of MCM-41-PNIPAM–linalool composite carriers with different AM doping ratios as a function of sustained-release time at same temperatures. (A) Release curves of MCM-41-PNIPAM–linalool composite carriers and linalool at 30 °C. (B) Release curves of MCM-41-PNIPAM–linalool composite carriers and linalool at 50 °C. (C) Release curves of MCM-41-PNIPAM–linalool composite carriers and linalool at 80 °C. A and D correspond to the samples with different AM doping ratios listed in Table 3, respectively.

At 30 °C (Figure 4A), the temperature was close to the LCST of Group A but remained well below that of Group D. Under these conditions, the polymer networks of both carriers were highly hydrated and maintained a swollen structure with relatively small pore channels, resulting in high diffusion resistance for linalool transport. Consequently, both composite carriers effectively suppressed the burst release observed for free linalool and exhibited a slow and sustained-release profile. Since the thermosensitive networks of Groups A and D remained in similar hydrated states at this temperature, no significant difference in their release behaviors was observed.

At 50 °C (Figure 4B), the temperature exceeded the LCST of Group A, inducing dehydration and contraction of the PNIPAM network. This phase transition reduced diffusion resistance and facilitated the outward diffusion of linalool, leading to a pronounced increase in the release rate. In contrast, the temperature remained below the LCST of Group D, allowing its polymer network to retain a hydrated and swollen structure that continued to restrict molecular diffusion. As a result, Group D maintained a relatively slow and stable release profile. The distinct release behaviors of the two groups at this temperature further demonstrate that adjusting the LCST effectively shifts the thermoresponsive release window.

At 80 °C (Figure 4C), the temperature exceeded the LCST values of both composite carriers, causing extensive dehydration and contraction of the thermosensitive polymer networks. Under these conditions, free linalool was released rapidly with virtually no sustained-release capability. Group A underwent substantial network collapse, markedly accelerating linalool diffusion and consequently reducing its sustained-release performance. In comparison, Group D exhibited a less pronounced increase in the release rate. This behavior is likely attributable to its higher LCST and the enhanced diffusion resistance provided by the MCM-41 mesoporous framework, which partially compensated for the reduction in polymer-network resistance after thermal collapse, thereby preserving sustained-release capability over prolonged release periods.

Overall, the release results obtained under gradient temperatures demonstrate that AM incorporation effectively increases the LCST of PNIPAM, thereby extending and tailoring its thermoresponsive range. More importantly, the synergistic combination of LCST modulation and the mesoporous confinement effect of MCM-41 enables the dynamic regulation of the polymer-network structure and molecular diffusion pathway in response to temperature variation, resulting in controllable temperature-responsive sustained release of linalool.

To further understand the release behavior of linalool, four kinetic models (zero-order, first-order, Higuchi, and Ritger–Peppas models) were employed to fit the release data obtained at different temperatures. The fitting curves are presented in Figure S6, and the corresponding kinetic parameters and R^2^ are summarized in Table 4. For pure linalool (30-L, 50-L, 80-L), both zero-order and Higuchi models exhibited poor fitting accuracy with *R^2^* generally lower than 0.85. In contrast, the first-order model achieved the highest fitting precision, which gradually improved with increasing temperature (30-L: *R* ^2^ = 0.99609; 50-L: *R* ^2^ = 0.99926; 80-L: *R* ^2^ = 0.99998). This indicates that the release rate of free linalool was directly dependent on the residual linalool concentration, demonstrating a typical concentration-driven instantaneous burst-release pattern without sustained-release capacity. The diffusion index *n* values obtained from the Ritger–Peppas model were all much lower than 0.45, further confirming that the release of pure linalool was dominated by rapid diffusion.^41^

**Table 4:** Kinetic Model Fitting Parameters of 30-A, 50-A, 80-A, 30-D, 50-D, 80-D, 30-L, 50-L, and 80-L^a^

	***M***_**0**_ **/mg**	**zero-order**	**First-order**	**Higuchi**	**Ritger**–**Peppas**
30-A	200	*K* = 0.00231	*K* = 0.17690	*K* = 0.05369	*K* = 0.06617 R^2^ = 0.99881
		*R* ^2^ = 0.93545	*R* ^2^ = 0.91062	*R* ^2^ = 0.97697	*n* = 0.27887
50-A		*K* = 0.00380	*K* = 0.21249	*K* = 0.03368	*K* = 0.10332 *R* ^2^ = 0.99625
		*R* ^2^ = 0.41725	*R* ^2^ = 0.94138	*R* ^2^ = 0.76202	*n* = 0.24397
80-A		*K* = 0.00776	*K* = 0.28130	*K* = 0.05184	*K* = 0.13975 *R* ^2^ = 0.99000
		*R* ^2^ = 0.67438	*R* ^2^ = 0.99213	*R* ^2^ = 0.90369	*n* = 0.21761
30-D	200	*K* = 0.00380	*K* = 0.13742	*K* = 0.02982	*K* = 0.06249 *R* ^2^ = 0.99701
		*R* ^2^ = 0.53743	*R* ^2^ = 0.91472	*R* ^2^ = 0.91867	*n* = 0.29424
50-D		*K* = 0.00422	*K* = 0.17722	*K* = 0.02936	*K* = 0.07774 *R* ^2^ = 0.99663
		*R* ^2^ = 0.75316	*R* ^2^ = 0.98654	*R* ^2^ = 0.92692	*n* = 0.23169
80-D		*K* = 0.00449	*K* = 0.16024	*K* = 0.03383	*K* = 0.07890 *R* ^2^ = 0.99901
		*R* ^2^ = 0.49494	*R* ^2^ = 0.89878	*R* ^2^ = 0.82924	*n* = 0.25569
30-L	200	*K* = 0.00998	*K* = 0.97388	*K* = 0.10319	*K* = 0.82648 *R* ^2^ = 0.95184
		*R* ^2^ = 0.12302	*R* ^2^ = 0.99609	*R* ^2^ = 0.40199	*n* = 0.05027
50-L		*K* = 0.00927	*K* = 0.97435	*K* = 0.09862	*K* = 0.91624 *R* ^2^ = 0.98542
		*R* ^2^ = 0.08247	*R* ^2^ = 0.99926	*R* ^2^ = 0.832623	*n* = 0. 01891
80-L		*K* = 0.00894	*K* = 0.97057	*K* = 0.09648	*K* = 0.91731 *R* ^2^ = 0.99858
		*R* ^2^ = 0.06468	*R* ^2^ = 0.99998	*R* ^2^ = 0.32623	*n* = 0.00299

a A and D represent samples with different AM/NIPAM ratios listed in Table 3, respectively, while L represents free linalool.

For the Group A thermoresponsive composite carriers (30-A, 50-A, 80-A), the Ritger–Peppas model provided the optimal fitting results among the four models, with *R* ^2^ values of 0.99881, 0.99625, and 0.99000, respectively. This suggests that linalool release was synergistically regulated by diffusion through the polymer gel network and mesoporous channels. At 30 °C, the 30-A sample showed a diffusion index *n* of 0.27887 (<0.45), indicating a Fickian diffusion-dominated release mechanism. At this temperature, the PNIPAM gel remained in a hydrated and swollen state with a dense network structure and high channel diffusion resistance, which enabled linalool to diffuse slowly through the tortuous pore channels. When the temperature increased to 50 °C (exceeding the LCST of Group A), the *n* value of 50-A decreased to 0.24397, still following Fickian diffusion. However, the Ritger–Peppas rate constant (*K*) increased from 0.06617 to 0.10332, implying that the thermoresponsive polymer underwent hydrophobic shrinkage, which expanded the gel channels, reduced diffusion resistance, and significantly accelerated the release rate. Further increasing the temperature to 80 °C led to a continuous increase in *K* to 0.13975 and a decrease in *n* to 0.21761, as the gel shrank more thoroughly to form more unobstructed diffusion channels and further promote linalool release.

Similarly, the Ritger–Peppas model best described the release behavior of Group D thermoresponsive composite carriers (30-D, 50-D, 80-D), with high *R* ^2^ values of 0.99701, 0.99663, and 0.99901, respectively. All D samples exhibited Fickian diffusion-dominated release, with *n* values ranging from 0.23 to 0.30 (all less than 0.45). At 30 and 50 °C, both temperatures were lower than the LCST of Group D, and the gel maintained a stable hydrated pore-blocking structure. Consequently, 30-D and 50-D displayed low *K* values (0.06249 and 0.07774, respectively) and steady release rates, achieving stable sustained-release performance. When the temperature reached 80 °C and exceeded the LCST of Group D, the gel underwent hydrophobic shrinkage, and the *K* of 80-D increased to 0.07890. Nevertheless, the increment was remarkably smaller than that of Group A, and the *n* value remained at a low level of 0.25569. These results demonstrate that Group D with higher AM doping content possessed lower thermosensitivity, enabling the gel network to retain a certain diffusion barrier effect at high temperatures and achieve more robust sustained-release performance.^41^

### 3.4 Evaluation of the Bactericidal Performance of the MCM-41-PNIPAM–Linalool Composite

The antibacterial activity of the prepared materials was evaluated by using the spread-plate method against three representative bacterial strains, including *S. aureus*, *E. coli*, and *B. subtilis*. The corresponding colony images are shown in Figure 5. The blank control, pure MCM-41, and MCM-41-PNIPAM groups exhibited comparable bacterial growth with dense colonies, indicating the negligible antibacterial effects of the carrier and polymer matrix under the tested conditions. In contrast, the MCM-41-PNIPAM–linalool composite showed a remarkable reduction in bacterial colonies, demonstrating strong antibacterial activity against all three strains. These results indicate that the antibacterial performance mainly originated from the loaded linalool, while MCM-41 and PNIPAM primarily served as mesoporous and thermoresponsive carriers for the controlled linalool delivery. The composite system effectively preserved the antibacterial activity of linalool and exhibited broad-spectrum antibacterial effects against both Gram-positive and Gram-negative bacteria.

**Figure 5:**
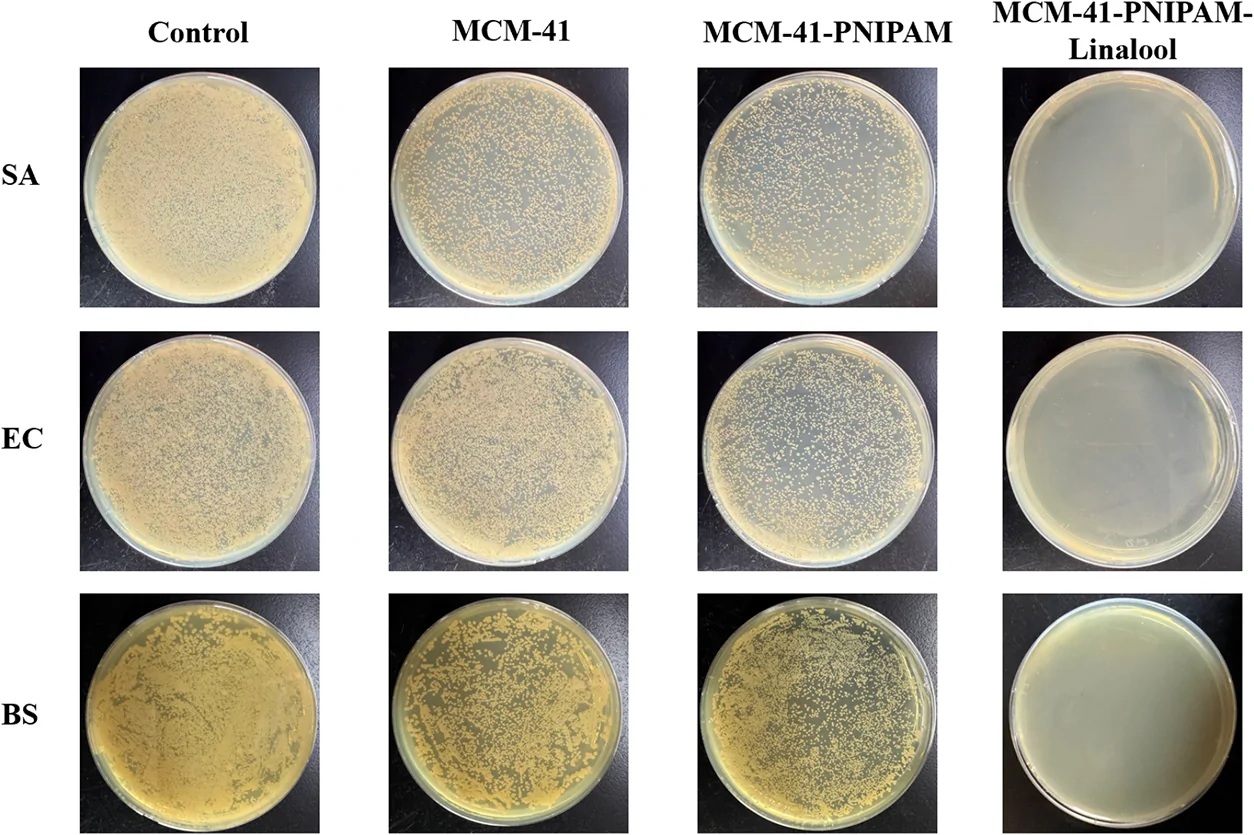
Photographs of bacterial colonies on agar plates after treatment with the blank control, MCM-41, MCM-41-PNIPAM, and MCM-41-PNIPAM–linalool composites against *S. aureus*, *E. coli*, and *B. subtilis*.

The UV and storage stability of the MCM-41-PNIPAM–linalool composite were evaluated by quantifying the retained linalool content after the respective treatments using UV–vis spectrophotometry, followed by assessing the antibacterial activity of the treated composites via a plate colony-counting assay. The corresponding results are summarized in Table 5 and Figure 6. After 1 day of UV irradiation, 17.54% of linalool remained in the composite, while 8.70% was retained after 15 days of dark storage at ambient temperature, indicating good UV resistance and storage stability. Furthermore, the aged composites maintained considerable antibacterial activity after both treatments, demonstrating the excellent functional stability of the linalool-loaded thermoresponsive carrier.

**Table 5:** Linalool Retention Rate of the MCM-41-PNIPAM–Linalool Composite after UV Irradiation and Storage at Room Temperature

**sample**	**UV**	**room temperature storage**
MCM-41-PNIPAM–linalool	17.54 ± 0.45%	8.70 ± 0.11%

**Figure 6:**
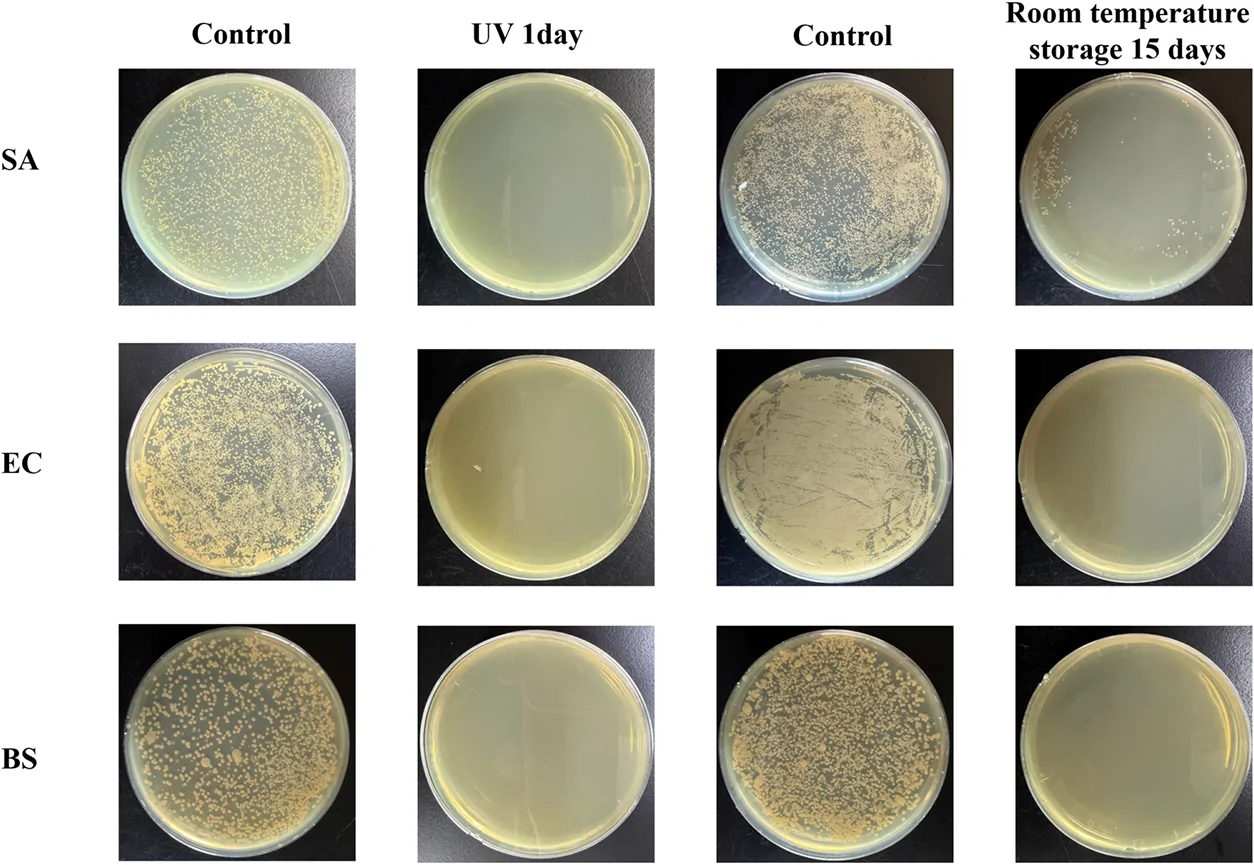
Colony images of *S. aureus*, *E. coli*, and *Bacillus subtilis* after treatment with MCM-41-PNIPAM–linalool composites subjected to UV irradiation for 1 day and dark storage at room temperature for 15 days. The untreated bacterial suspension without composite addition was used as the control group.

To evaluate the antibacterial activity of MCM-41-PNIPAM–linalool, the disk diffusion method was used to measure the inhibition zone diameters against the tested bacterial strains. As shown in Figure S7A, at a concentration of 50 mg/mL, the inhibition zone diameters against *S. aureus*, *E. coli*, and *B. subtilis* were 3.54 ± 0.12, 3.61 ± 0.08, and 4.23 ± 0.10 cm, respectively. No inhibition zone was observed around the control sample (blank filter paper without material loading). These results indicate that the material exhibits significant broad-spectrum antibacterial activity against all three tested bacterial strains.

Furthermore, to quantitatively assess the antibacterial efficacy of linalool, we selected *B. subtilis* as the model strain. The minimum inhibitory concentration (MIC) and minimum bactericidal concentration (MBC) were determined using the broth microdilution method combined with a plate colony-counting assay. As shown in Figure S7B,C, the MIC and MBC of the material against *B. subtilis* were 2 and 8 mg/mL, respectively. These results demonstrate that the material can effectively inhibit bacterial growth at relatively low concentrations while exerting bactericidal effects at higher concentrations.

CLSM combined with live/dead staining was performed to visualize the temperature-responsive antibacterial activity of the MCM-41-PNIPAM–linalool composite (Figure 7). The Control, D-40 °C, and A-25 °C groups exhibited predominant green fluorescence, indicating high bacterial viability under conditions below the LCST. In contrast, the A-40 °C group showed markedly increased red fluorescence due to PNIPAM phase transition and enhanced linalool release at temperatures above the LCST. These results demonstrate that the composite possesses temperature-dependent antibacterial activity and enables thermally triggered antibacterial responses.

**Figure 7:**
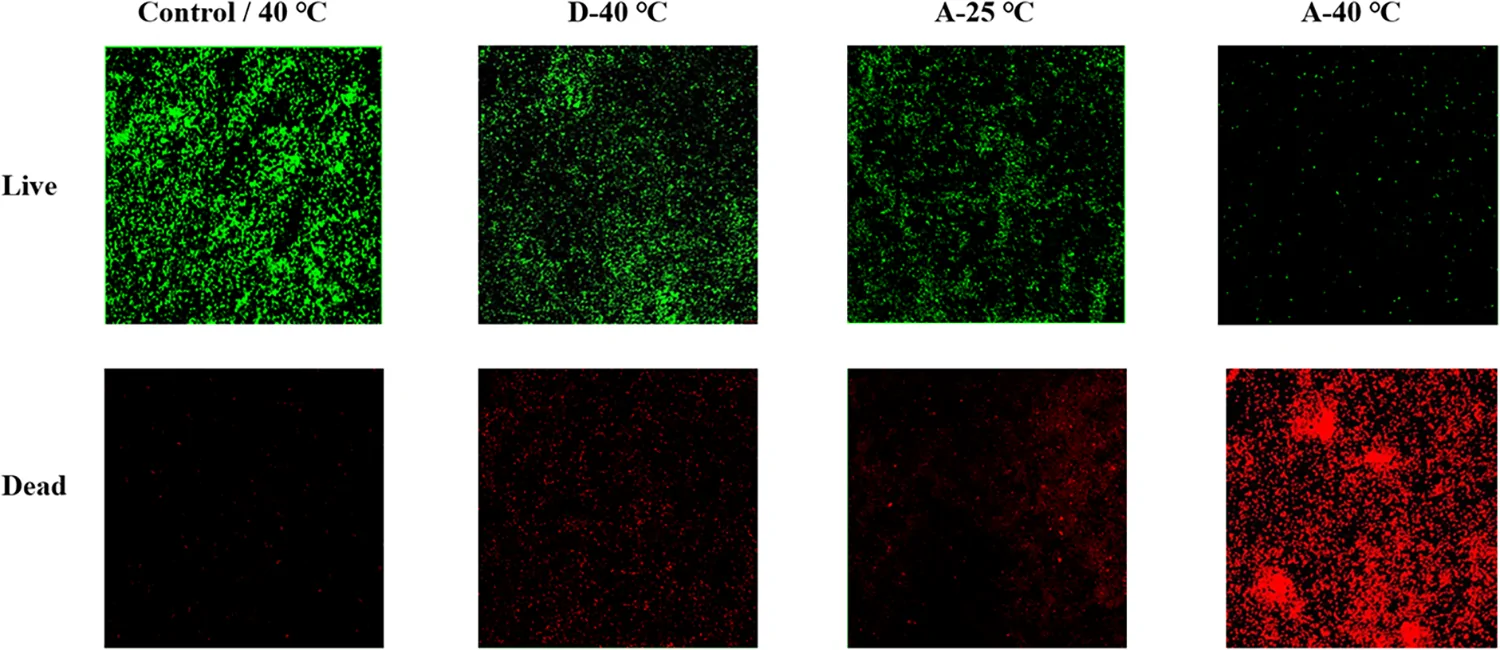
CLSM of bacterial viability after treatment with different temperature-carrier combinations. The upper row shows live bacteria labeled with SYTO 9 (green fluorescence), and the lower row shows dead bacteria labeled with propidium iodide (PI) (red fluorescence). A and D correspond to the samples with different AM doping ratios in Table 3, respectively.

Furthermore, the release profiles of MCM-41-PNIPAM–linalool and free linalool were quantitatively investigated after 3 h of release at different temperatures, with the results summarized in Table 6. As the temperature increased, MCM-41-PNIPAM–linalool exhibited a gradual enhancement in linalool release, achieving release rates of 9.1, 14.4, and 18.3% at 30, 50, and 80 °C, respectively. In comparison, free linalool showed an uncontrolled burst-release behavior, with release rates reaching 89.5, 94.9, and 97.4% under identical conditions. To further correlate the temperature-responsive release behavior with antibacterial performance, plate colony assays were conducted using linalool concentrations equivalent to the amounts released at different temperatures (Figure S8). Colony quantification using ImageJ Pro confirmed that the enhanced linalool release triggered by elevated temperatures effectively improved the antibacterial activity of the composite system.

**Table 6:** Release Amount of Linalool and Corresponding Bacteriostatic Rate of MCM-41-PNIPAM–Linalool at Different Temperatures

**Sample**	**temperature (°C)**	**release mass (%)**	**bacteriostatic rate (%)**
MCM-41-PNIPAM–linalool	30.0	9.1	19.3
	50.0	14.4	81.2
	80.0	18.3	97.8
linalool	30.0	89.5	100.0
	50.0	94.9	100.0
	80.0	97.4	100.0

### 3.5 Biocompatibility of the MCM-41-PNIPAM Composite

The cytocompatibility of the MCM-41-PNIPAM composite was evaluated using L929 fibroblasts with untreated cells serving as the control group. Cell morphology observations and CCK-8 assays were performed after exposure to extracts of MCM-41and MCM-41-PNIPAM. The results are shown in Figure 8A,B. All groups exhibited well-spread cells with clear boundaries, and no apparent morphological changes were observed compared with the control group. Furthermore, the CCK-8 assay showed that cell viabilities remained above 97% for all material-treated groups with no significant differences from the control. These results demonstrate that the developed composite possesses an excellent cytocompatibility and negligible cytotoxicity.

**Figure 8:**
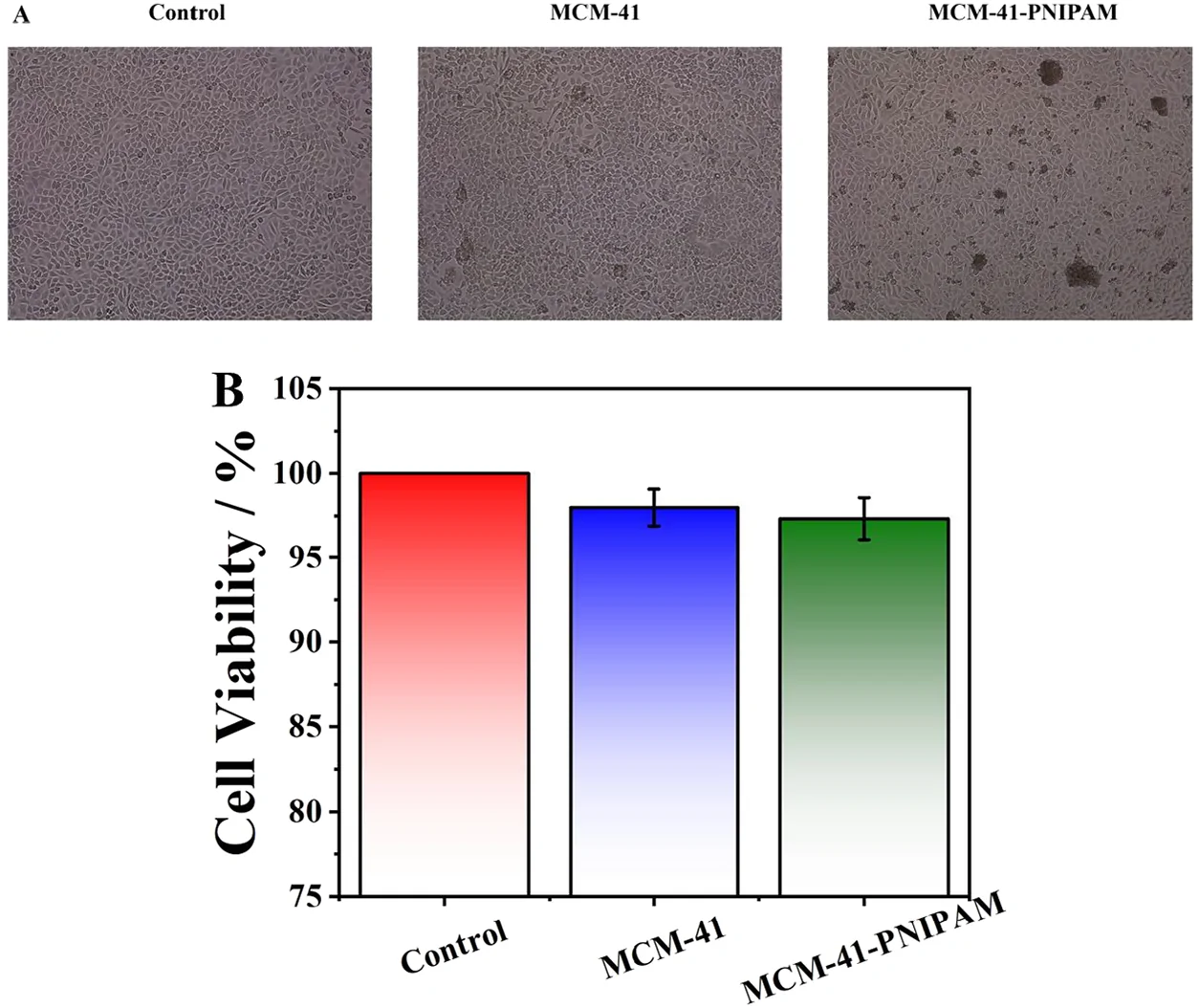
Cell morphology and viability of L929 mouse fibroblasts after incubation with extracts of different materials for 2 days. (A) Bright-field optical micrographs of cells treated with Control, MCM-41, and MCM-41-PNIPAM extracts. (B) Cell viability determined by the CCK-8 assay after 2 days of incubation.

## Conclusion

In this study, two types of mesoporous silica, SBA-15 and MCM-41, were successfully synthesized, and MCM-41 with superior loading compatibility was selected as the carrier. Through surface functional modification, copolymer grafting, and linalool loading, a P­(NIPAM-SiO_2_-AM-BIS-linalool) thermosensitive sustained-release composite carrier was fabricated. Characterization confirmed successful thermosensitive polymer conjugation and an effective linalool loading.

The incorporation of hydrophilic monomer AM enabled precise LCST modulation of the composite carrier. The LCST increased progressively from 31 - 33 °C to 82–85 °C with higher AM content. Sustained-release studies revealed that the carrier exhibited thermosensitive release characteristics, where linalool release rate surged above the LCST, and AM content synergistically modulated release behavior. Antibacterial experiments verified the temperature-gated bactericidal efficiency of the composite carrier, which could effectively inhibit the proliferation of *B. subtilis*, *S. aureus*, and *E. coli* after temperature-triggered linalool release.

The synthetic strategy proposed in this study provides an effective approach to overcoming the drawbacks of high volatility and poor stability of linalool. The prepared thermosensitive sustained-release composite carrier offers new materials and a general method for the construction of intelligent fresh-keeping systems and is expected to be further extended to the fields of nanoparticle modification and diversified thermosensitive functional materials.

## Supplementary Material



## Notes

Visual appearances of SBA-15 and MCM-41 carriers with different linalool loadings (Figure S1); SEM images of MCM-41 and MCM-41-P­(NIPAM-AM-BIS)–linalool composite microspheres (Figure S2); nitrogen adsorption–desorption isotherms and pore size distributions of MCM-41, MCM-41-P­(NIPAM-AM-BIS), and their linalool-loaded composites (Figure S3); UV–vis absorption spectra of linalool standard solutions and the corresponding calibration curve (Figure S4); DSC curves of PNIPAM–SiO_2_-linalool composite carriers with different AM doping ratios (Figure S5); time-dependent cumulative release profiles of different samples fitted by four kinetic models (Figure S6); antibacterial activity of the samples determined by the disk diffusion method, broth dilution method, and agar plate counting (Figure S7); and colony morphology of bacteria treated with free linalool or linalool released from MCM-41-PNIPAM–linalool at equivalent concentrations (Figure S8) (PDF)
