# Supplementary material for: Smart Thermosensitive Mesoporous Silica Composites for Precisely Triggered Linalool Release
Source: ACS Omega. 2026 Oct 28;11(44):68329–40. doi: 10.1021/acsomega.6c04870 (PMC13757083; doi:10.1021/acsomega.6c04870)
Supplement: Supplementary file 1 [file ao6c04870_si_001.pdf]

# Smart Thermosensitive Mesoporous Silica Composites for Precisely Triggered Linalool Release

Zhenhui Gao [a], Yanhua Lu [b], Fengfeng Zhang [b,*], Wenlong Bao [b], Xinhua Song [b], Fanyu Kong [a], Rongning Liang[c] , Dean Song [a,*]

a Tobacco Research Institute, Chinese Academy of Agricultural Sciences, Qingdao 266101, China

b China Tobacco Shandong Industrial Co., Ltd., Jinan 250014, China

c Shandong Key Laboratory of Coastal Zone Environmental Processes and Ecological Security, Yantai Institute of Coastal Zone Research, Chinese Academy of Sciences, Yantai, Shandong 264003, P. R. China

* E-mail addresses: songdean@caas.cn

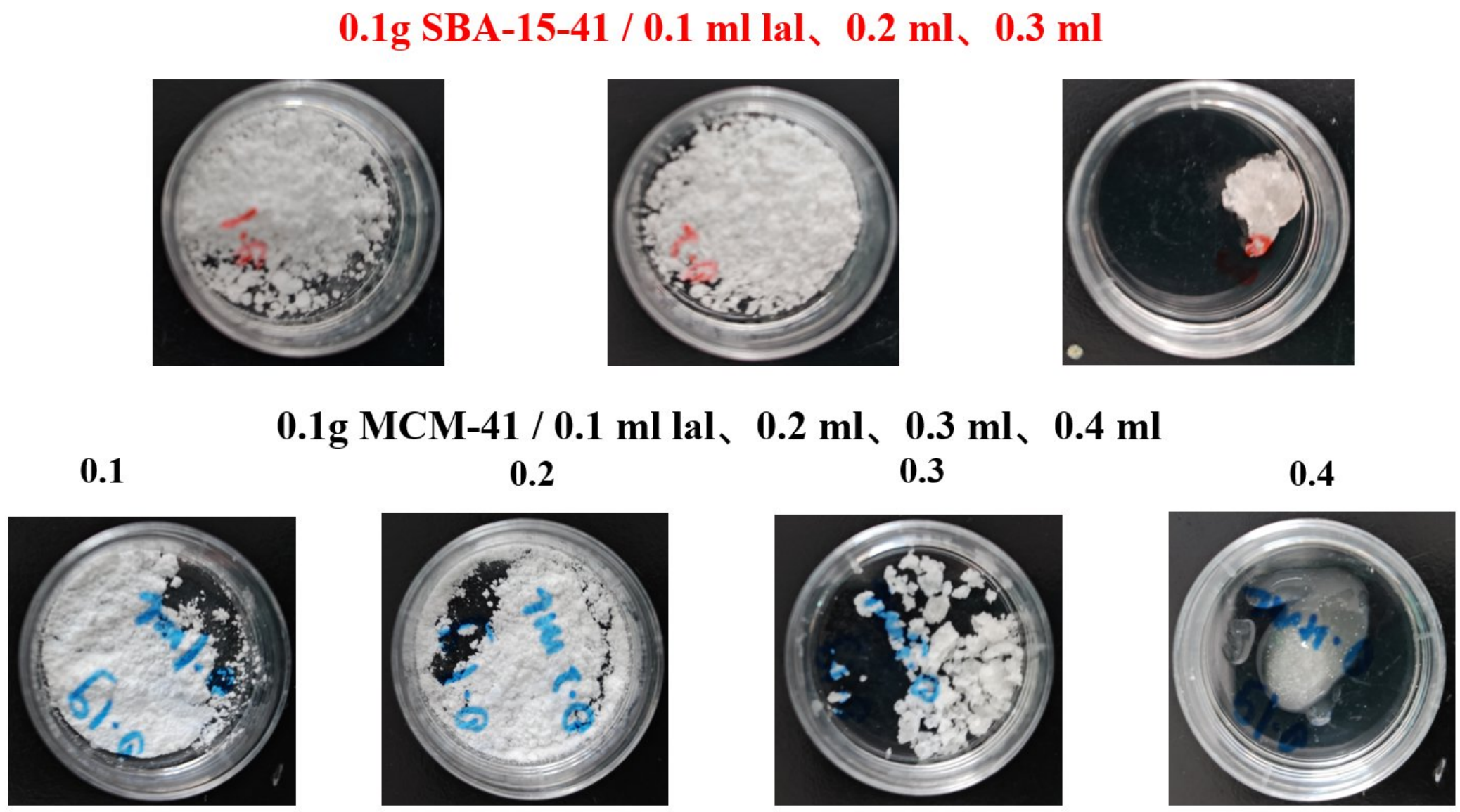


**Figure S1.** Visual appearances of SBA-15 and MCM-41 carriers with varying loadings of linalool.

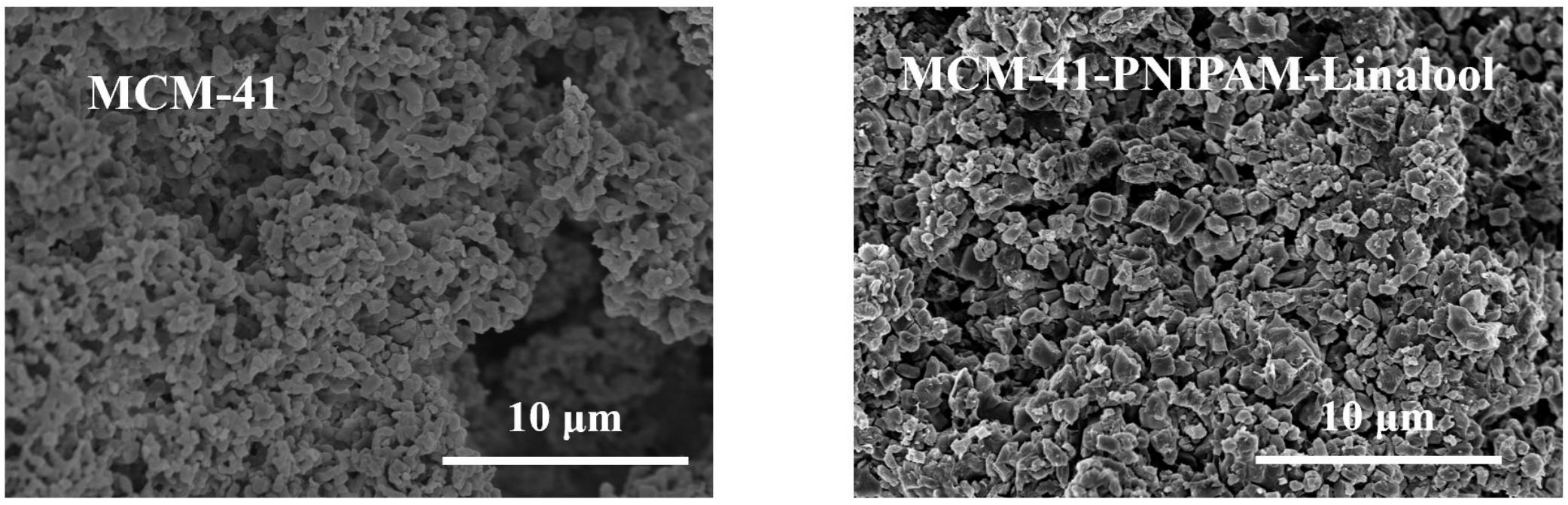


**Figure S2.** Comparison of SEM morphologies of MCM-41 and its copolymerized composite microspheres. The left panel shows the SEM image of MCM-41 mesoporous silica, while the right panel displays the SEM image of MCM-41-P(NIPAM-AM-BIS)-Linalool

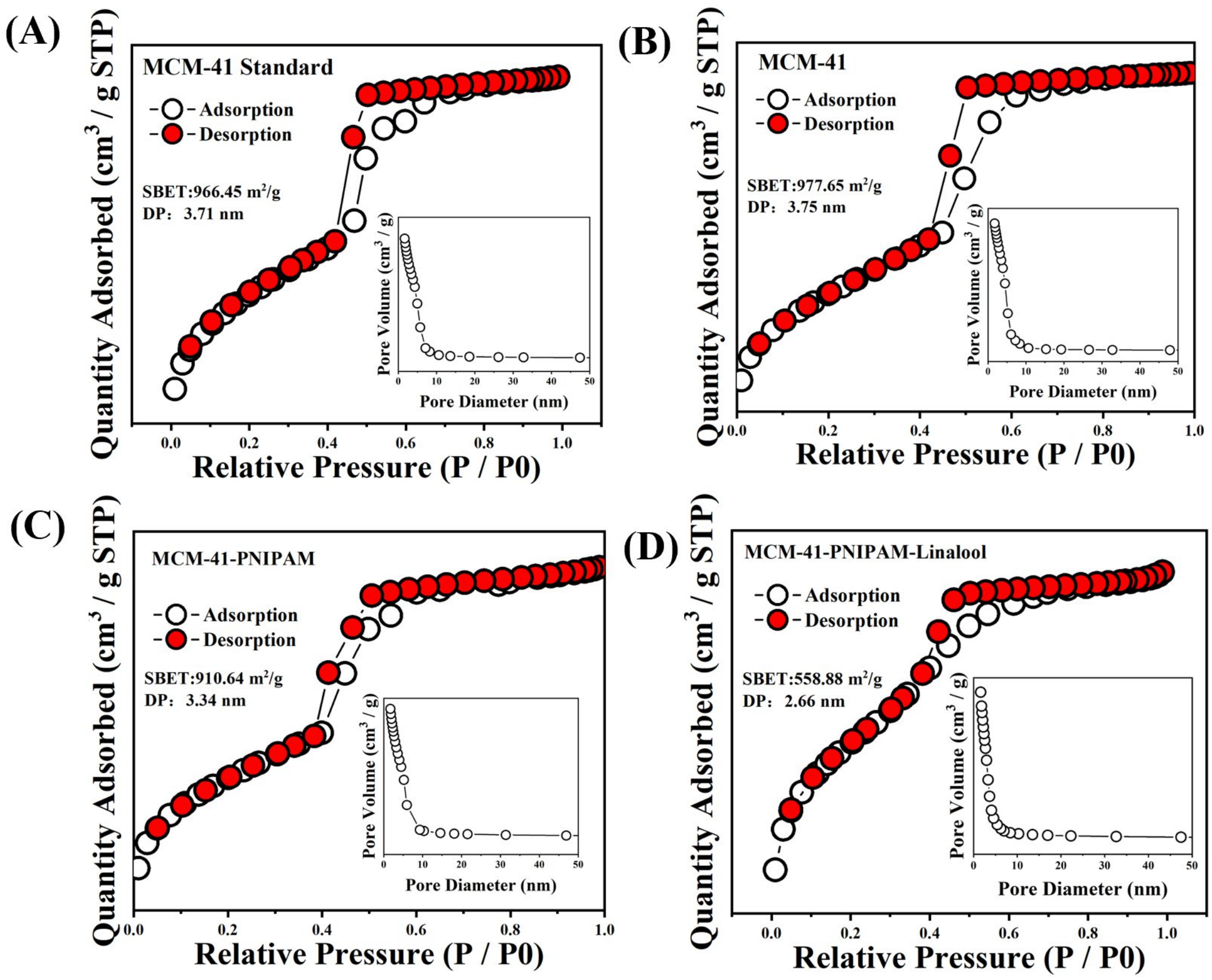


**Figure S3.** Nitrogen adsorption–desorption isotherms and pore size distributions of the as-synthesized composites were characterized. (A). MCM-41 Standard, (B). MCM-41, (C). MCM-41-P(NIPAM-AM-BIS), (D). MCM-41-P(NIPAM-AM-BIS)-Linalool.

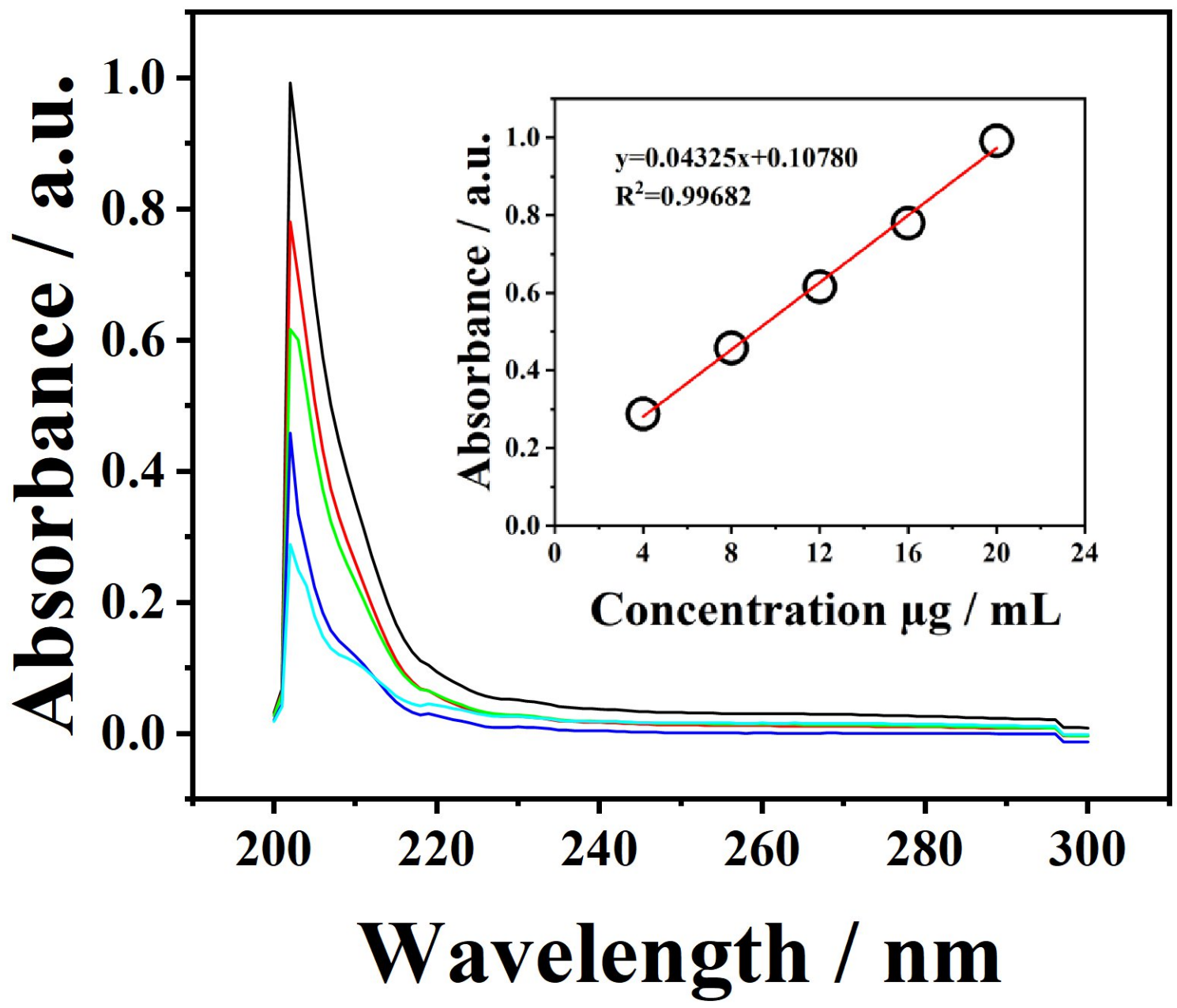


**Figure S4.** UV–vis absorption spectra of linalool standard solutions at different concentrations and the corresponding calibration curve (inset). The linear regression equation was y = 0.04325x + 0.10780 with a $R^2$ value of 0.99682.

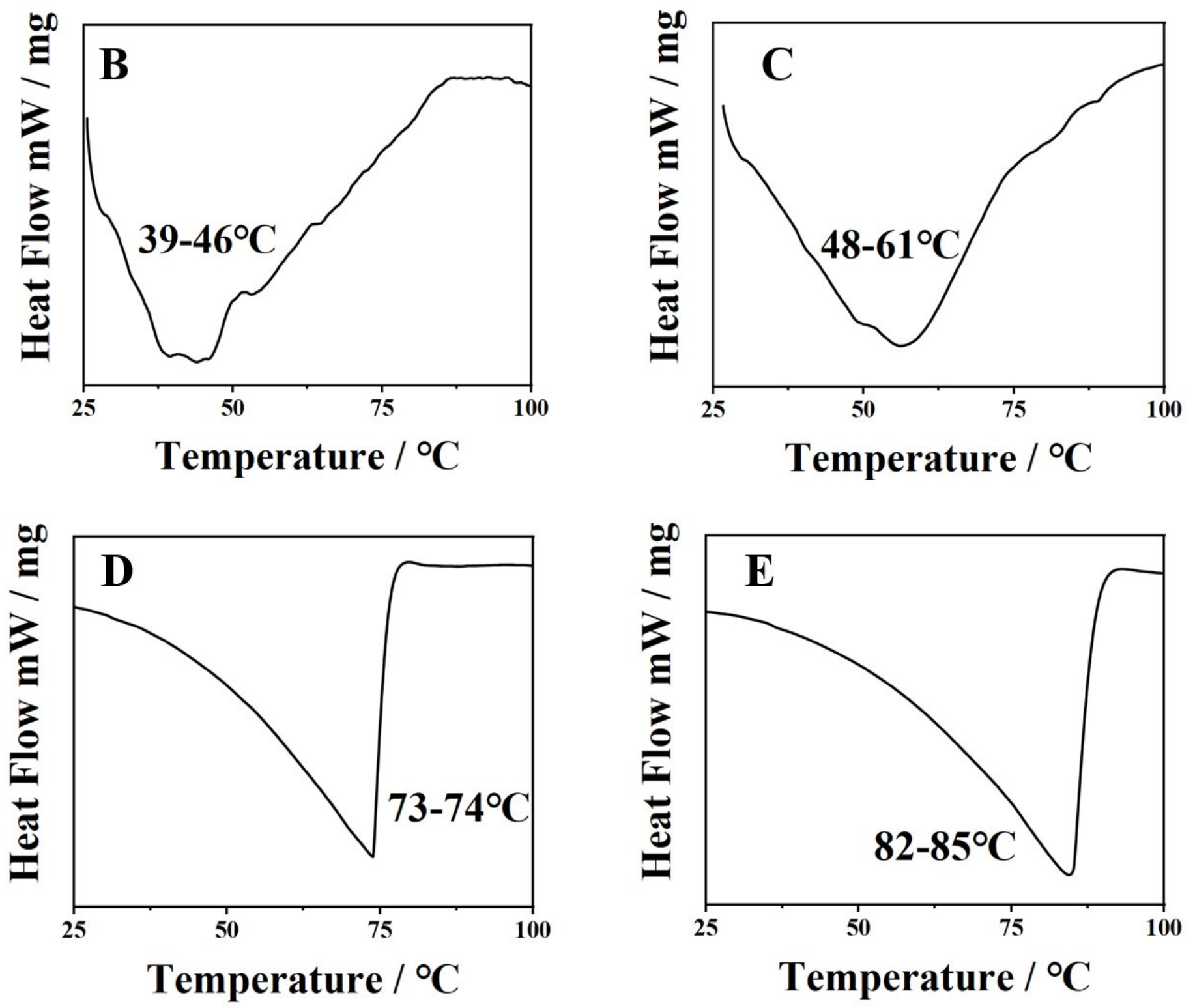


**Figure S5.** DSC curves of PNIPAM-$SiO_2$-linalool composite carriers with different AM doping ratios. B, C, D and E correspond to the samples with different AM doping ratios listed in **Table 3**, respectively.

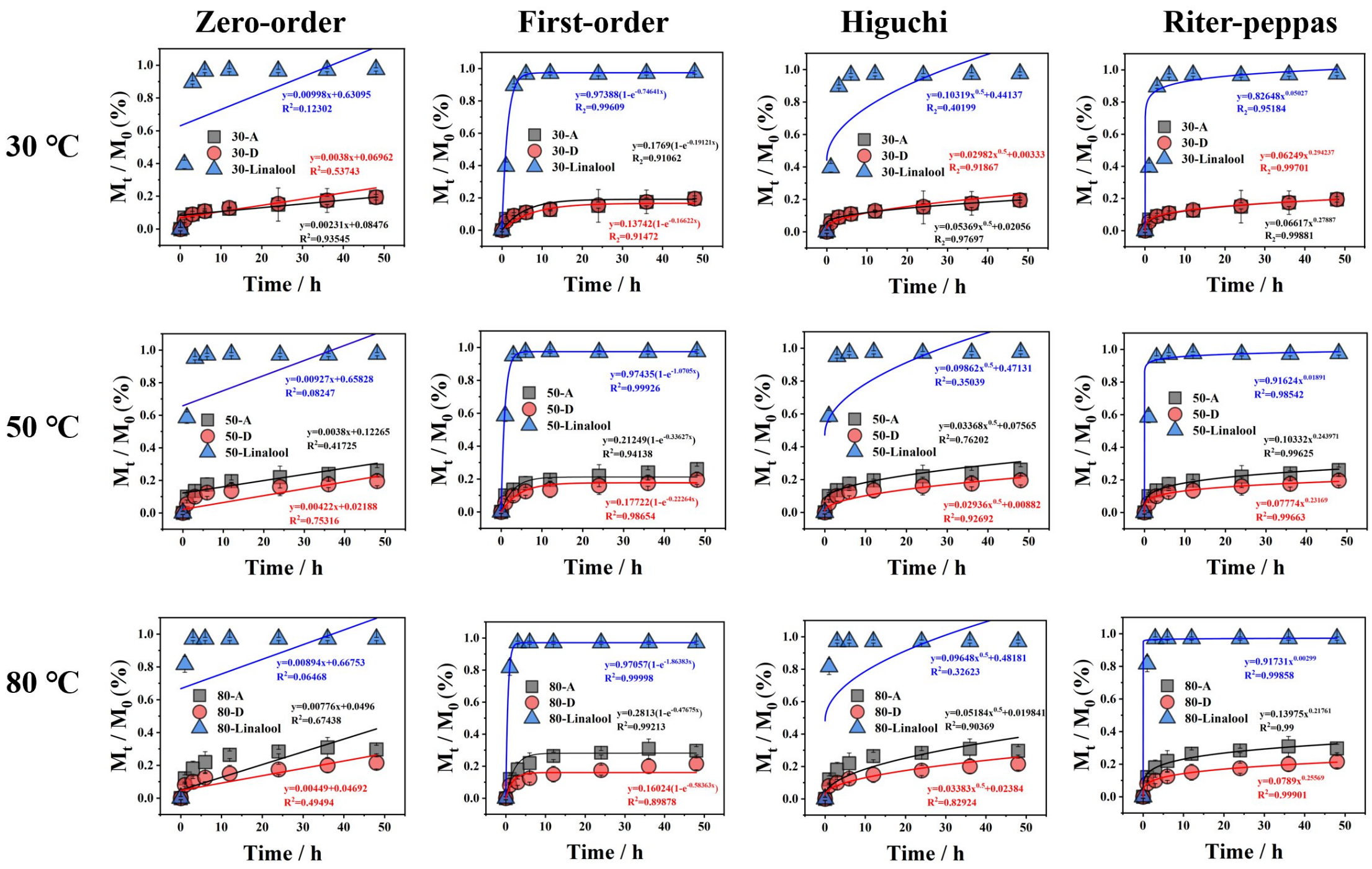


**Figure S6.** Time-dependent cumulative release profiles ($M_t/M_0$) of different samples (30/50/80-A, 30/50/80-D and 30/50/80-Linalool) fitted by four kinetic models (linear model, first-order kinetic model, Higuchi model and Ritger-Peppas model from left to right columns) at corresponding conditions; the fitting equations and coefficients of determination $R^2$ are displayed in each subgraph.

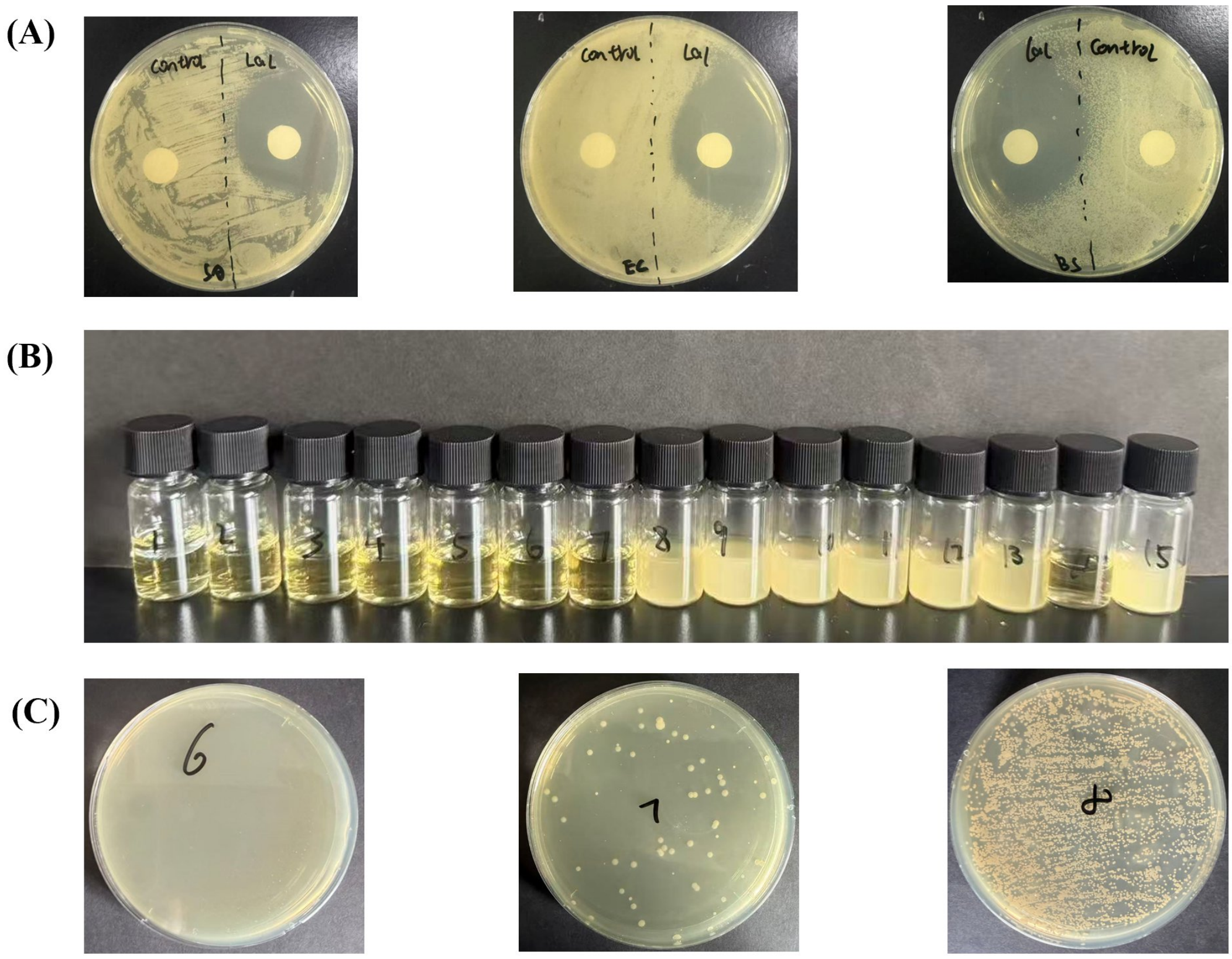


Figure S7. Determination of antibacterial activity of the sample. (A) Determination of inhibition zones by the disk diffusion method. SA: *Staphylococcus aureus*; EC: *Escherichia coli*; BS: *Bacillus subtilis*. Lal represents the experimental group, and Control represents the control group. (B) Gradient culture system established by two-fold broth dilution method. The concentrations of sample vials 1-13 ranged from 256 mg/mL to 0.0625 mg/mL. Vial 14 was the blank control with pure medium, and vial 15 served as the positive growth control containing medium and bacterial suspension. (C) Colony growth on agar plates after gradient dilution of different treatment groups. Plates 6, 7 and 8 correspond to MIC test groups at concentrations of 8 mg/mL, 4 mg/mL and 2 mg/mL, respectively.

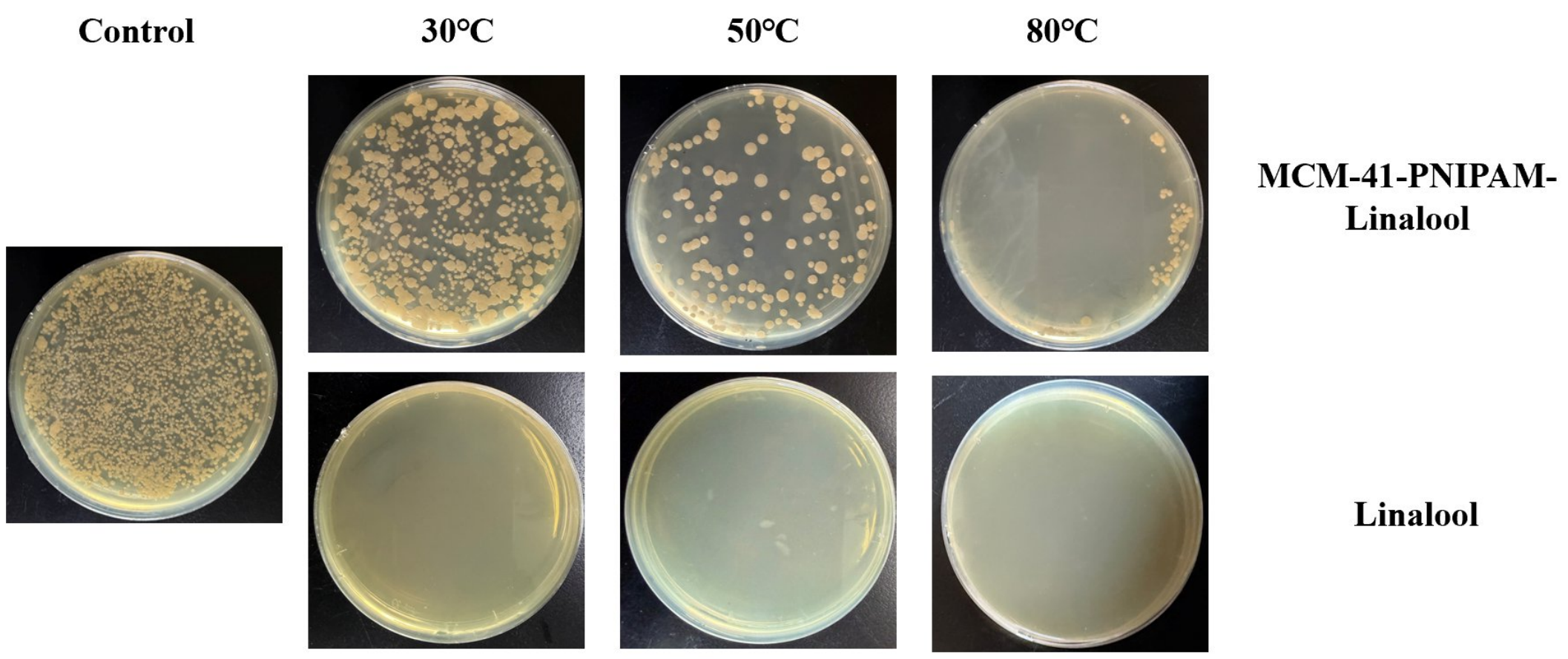


**Figure S8.** Colony morphology of bacteria in the blank control group and groups treated with free linalool or linalool released from MCM-41-PNIPAM-Linalool at equivalent concentrations. The linalool concentrations were determined based on the release amounts of MCM-41-PNIPAM-Linalool and free linalool at 30, 50, and 80℃, as provided in Table 6.
